# A *galU *mutant of *francisella tularensis *is attenuated for virulence in a murine pulmonary model of tularemia

**DOI:** 10.1186/1471-2180-11-179

**Published:** 2011-08-05

**Authors:** Himangi R Jayakar, Jyothi Parvathareddy, Elizabeth A Fitzpatrick, Xiaowen R Bina, James E Bina, Fabio Re, Felicia D Emery, Mark A Miller

**Affiliations:** 1 Department of Microbiology, Immunology, and Biochemistry, The University of Tennessee Health Science Center, 858 Madison Avenue, Memphis, Tennessee 38163, USA; 2 Department of Immunology and Microbial Sciences, The Scripps Research Institute, 10550 North Torrey Pines Road, La Jolla, CA 92037, USA

## Abstract

**Background:**

A number of studies have revealed that *Francisella tularensis *(FT) suppresses innate immune responses such as chemokine/cytokine production and neutrophil recruitment in the lungs following pulmonary infection via an unidentified mechanism. The ability of FT to evade early innate immune responses could be a very important virulence mechanism for this highly infectious bacterial pathogen.

**Results:**

Here we describe the characterization of a *galU *mutant strain of FT live vaccine strain (LVS). We show that the *galU *mutant was highly attenuated in a murine model of tularemia and elicited more robust innate immune responses than the wild-type (WT) strain. These studies document that the kinetics of chemokine expression and neutrophil recruitment into the lungs of mice challenged with the *galU *mutant strain are significantly more rapid than observed with WT FT, despite the fact that there were no observed differences in TLR2 or TLR4 signaling or replication/dissemination kinetics during the early stages of infection. We also show that the *galU *mutant had a hypercytotoxic phenotype and more rapidly induced the production of IL-1β following infection either *in vitro *or *in vivo*, indicating that attenuation of the *galU *mutant strain may be due (in part) to more rapid activation of the inflammasome and/or earlier death of FT infected cells. Furthermore, we show that infection of mice with the *galU *mutant strain elicits protective immunity to subsequent challenge with WT FT.

**Conclusions:**

Disruption of the *galU *gene of FTLVS has little (if any) effect on *in vivo *infectivity, replication, or dissemination characteristics, but is highly attenuating for virulence. The attenuated phenotype of this mutant strain of FT appears to be related to its increased ability to induce innate inflammatory responsiveness, resulting in more rapid recruitment of neutrophils to the lungs following pneumonic infection, and/or to its ability to kill infected cells in an accelerated fashion. These results have identified two potentially important virulence mechanisms used by FT. These findings could also have implications for design of a live attenuated vaccine strain of FT because sublethal infection of mice with the *galU *mutant strain of FTLVS promoted development of protective immunity to WT FTLVS.

## Background

*Francisella tularensis *(FT) is a gram-negative intracellular bacterium that is the causal agent of tularemia. The *Francisellaceae *family of bacteria has a single genus, *Francisella*, which has been divided into two species: 1) *Francisella philomiragia *(a muskrat pathogen) and 2) *Francisella tularensis. Francisella tularensis *is further subdivided into four subspecies: *tularensis *(type A), *holarctica *(type B), *novicida*, and *mediasiatica *[1]. Of these, only subsp. *tularensis *and subsp. *holarctica *cause disease in humans [2]. FT *tularensis *is considered a prime candidate for use as a biological weapon because it is relatively easy to propagate and disseminate via aerosolization and because of the high morbidity and mortality associated with aerosol infection (LD_50_<10 CFU) [3,4]. The live vaccine strain (FT LVS), which was derived from FT *holarctica*, is only moderately virulent in humans [5] and retains virulence in mice. Because LVS causes an infection in mice that is similar to the human form of disease, the murine FT LVS infection model serves as an appropriate animal model of human tularemic disease [6-8].

FT is well adapted for growth and survival within host macrophages, as evidenced by its ability to inhibit phagosome/lysosome fusion and the respiratory burst, and to escape from the phagosome and replicate within the macrophage cytoplasm [9-11]. Moreover, it has been reported that the virulence of FT depends on its ability to escape into the host cytoplasm [10,12,13]. However, like many other successful pathogens, the key to the pathogenesis of FT may be in its ability to overcome, evade, and/or suppress innate host immune responses. For instance, FT is known to be relatively resistant to cationic antimicrobial peptides (CAMPs), which may in part be responsible for its ability to overcome host innate immunity [14,15]. In fact, it has been shown that FT mutant strains that are CAMP-sensitive are attenuated for virulence in mice [16,17]. FT is also able to evade (in part) innate immune detection because its lipopolysaccharide (LPS) has unusual modifications that render it immunologically inert and unable to stimulate TLR4 [17-19]. Indeed, FT *novicida *mutants that lacked these modifications and produced TLR4-stimulating LPS were able to induce stronger proinflammatory cytokine production and host innate responses resulting in rapid clearance and an attenuated phenotype in mice [17,20]. FT also appears to actively suppress acute inflammatory responses at early times after infection in lungs by a mechanism that has not yet been defined [21]. Following pulmonary infection of mice with FT, there is an initial lag in recruitment of neutrophils as well as a minimal proinflammatory cytokine response in the first 24-48 hours following infection with FT [22,23]. This quiescent period is typically followed by a massive neutrophil influx and profound upregulation of cytokine production that appears to contribute to FT pathogenesis [15,24,25]. The ability of WT FT to delay recruitment of neutrophils appears to be a critical virulence mechanism because FT mutants that fail to delay influx of neutrophils are rapidly cleared from the host and are attenuated for virulence [17,20]. Additionally, pretreatment of mice with rIL-12 resulted in early neutrophil recruitment to lungs and rapid immune clearance following infection with WT FT [26]. These data suggest that the kinetics, rather than the magnitude, of neutrophil recruitment at the site of infection are important for resolution of FT infection.

The efficacy of innate immune responses is largely dependent on interactions between host pattern recognition receptors with cell envelope components of the invading pathogen. Because WT FT appears to utilize undefined mechanism(s) to modulate innate immune signaling events to gain a survival advantage in mammalian hosts, we postulated that mutations that altered the cell envelope structure of FT would attenuate the virulence of the bacterium. In this report we have tested the hypothesis that *galU *is required for FT pathogenesis. The *galU *gene (FTL_1357) encodes for the production of UTP-glucose-1-phosphate uridyl transferase (or alternatively UDP-glucose pyrophosphorylase), an enzyme that catalyzes the formation of UDP-glucose from glucose-1-phosphate and UTP and is known to have a key role in biosynthesis of cell-envelope-associated carbohydrates (e.g. LPS and capsule) in a variety of bacteria [27-32]. The findings reported here revealed that disruption of the FT *galU *gene was highly attenuating *in vivo*, and that the reduction in virulence correlated with changes in the kinetics of chemokine production and neutrophil recruitment into the lungs following pulmonary infection. The *galU *mutant strain induced more rapid production of IL-1β *in vivo *and *in vitro *and it displayed a hypercytotoxic phenotype. We also found that mice that survived infection with the FT *galU *mutant strain developed protective immunity to subsequent challenge with WT FT.

## Results

### Effect of *galU *mutation on growth and intracellular survival of FT *in vitro*

**Figure 1:**
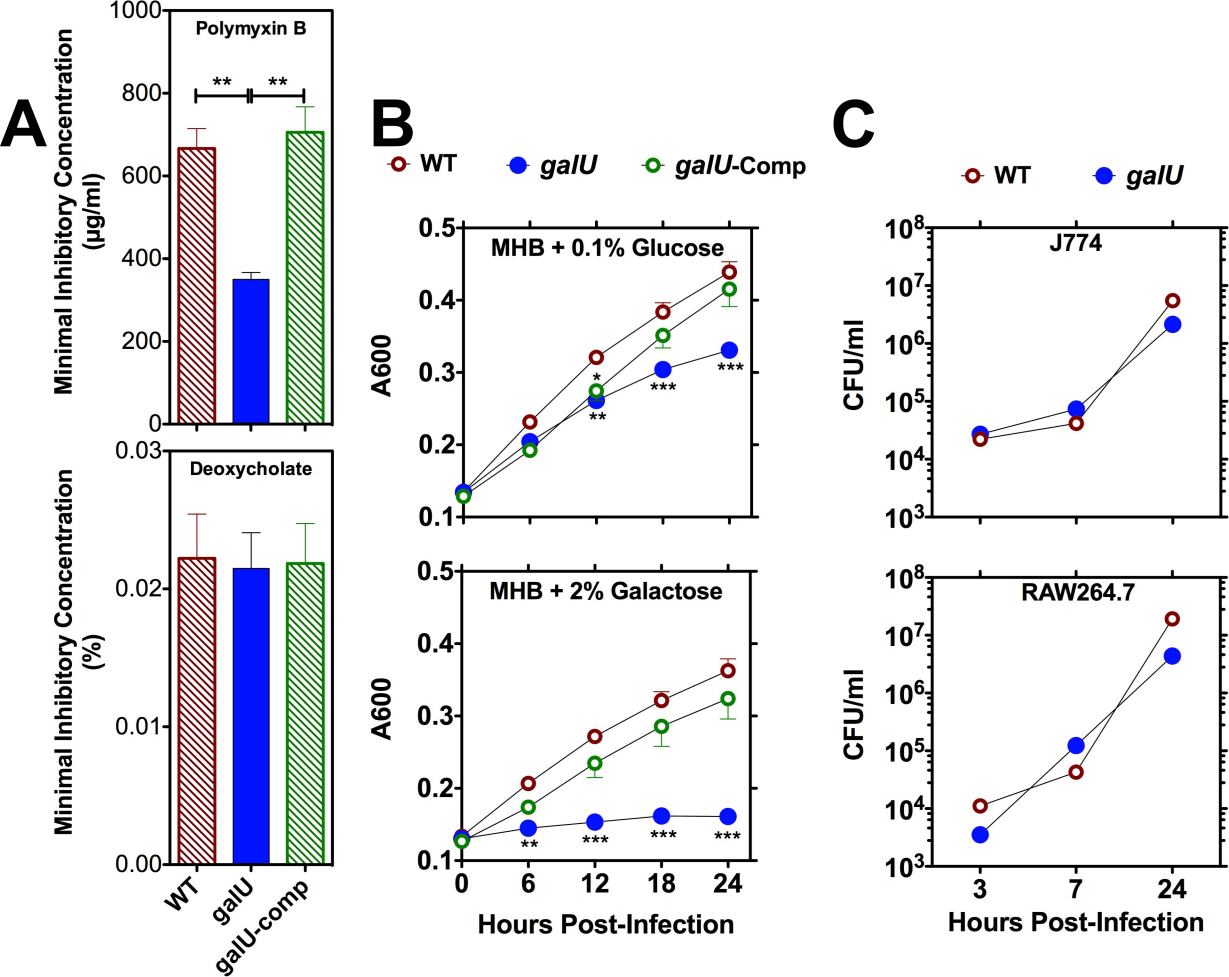
**Growth kinetics of the *galU *mutant *in vitro***. Growth of wild-type, *galU *mutant, and *galU*-complemented strains of FT after 48 hrs of culture was measured by the gradient plating technique to determine their sensitivity to polymyxin-B and deoxycholate. All data points represent the mean (^± ^SEM) of triplicate samples. Statistical analyses were performed via one-way ANOVA with Bonferroni post-tests. Statistically significant differences are indicated as follows: P < 0.01 (**) (**Panel A**). Growth of each strain cultured in MHB supplemented with either 0.1% glucose or 2% D-galactose (**Panel B**) or within macrophage-like murine cell lines (J774 or RAW264.7 at an MOI of 10, **Panel C**) was monitored over a 24 hour period. All data points represent the mean (^± ^SEM) of triplicate samples. Each panel is representative of at least three experiments of similar design. Statistical analyses were performed via two-way ANOVA with Bonferroni post-tests. Statistically significant differences are indicated as follows: P < 0.01 (**) and P < 0.001 (***).

The *galU *gene is highly conserved among the three major subspecies of FT (100% identity between *galU *genes of SchuS4 and LVS, 98.5% identity shared between FT *novicida *and LVS). In gram-negative bacteria, *galU *is typically part of an operon that is involved in galactose utilization and in the production of various exopolysaccharides [27,30,31]. The *galU *mutant strain characterized here was isolated from a random transposon library of FT LVS and was isolated as a polymyxin B hypersensitive strain (Figure 1A). The increased sensitivity of this *galU *mutant strain to cationic antimicrobials does not appear to be due to generalized outer envelope disintegrity because the mutant bacterium does not exhibit hypersensitivity to deoxycholate (an anionic bile acid) (Figure 1A) or the antibiotics chloramphenicol or tetracycline (data not shown).

The galU gene product is also known to be involved (but not required) in the catabolism of glucose and is required for the catabolism of galactose in bacteria and yeast [31,33,34]. Therefore, we predicted that the *galU *mutant strain would display a mild growth defect in minimal medium containing glucose as a sole sugar source, and would have a more marked growth defect when cultured in medium containing galactose as a sole source of sugar. To determine whether the *galU *mutant had a galactose utilization phenotype, we characterized its growth in Mueller-Hinton broth (MHB) supplemented with either glucose or galactose as a sole sugar source (it is important to note that our standard medium for culture of FT is MHB supplemented with 0.1% glucose as the sole source of sugar). As predicted, the *galU *mutant strain of FT displayed a mild growth defect in MHB supplemented with glucose and a severe growth defect in MHB supplemented with galactose. Complementation of the *galU *mutation restored WT growth kinetics in MHB supplemented with either glucose or galactose (Figure 1B ).

To determine if mutation of the *galU *gene resulted in an intracellular growth defect, we evaluated the ability of the WT and *galU *mutant strains of FT to grow within murine macrophage-like cells *in vitro*. The replication kinetics of the *galU *mutant within J774 or RAW 264.7 cells were indistinguishable from those of the WT strain (Figure 1C), indicating that mutation of the *galU *gene had no effect on uptake or intracellular survival/replication of the bacterium.

### Virulence of the *galU *mutant *in vivo*

**Figure 2:**
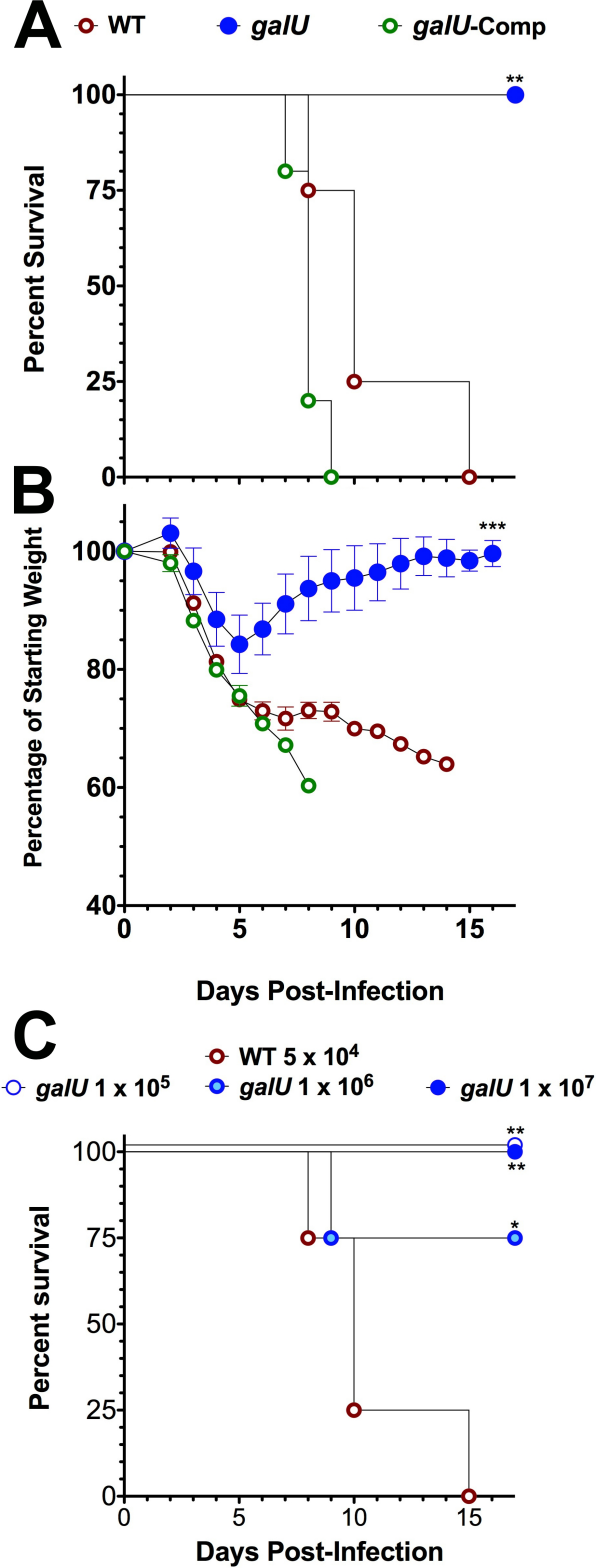
**Mutation of the *galU *gene attenuates virulence of FT**. C57BL/6 mice were infected intranasally with 5 × 10^4 ^CFU of WT (n = 9), the *galU *mutant (n = 10), or the *galU*-complemented strain (n = 5) strain of FTLVS, and their survival (**Panel A**) and weight (**Panel B**) were monitored. Statistical analyses of survival curves was performed using Gehan-Breslow-Wilcoxon tests and a p value of 0.005 is indicated (**). Statistical analysis of body weight retention was performed via one-way ANOVA with a Bonferroni multiple comparisons post-test and a p value of <0.0001 is indicated (***). **Panel C**: Survival was also monitored in C57Bl/6J mice challenged with a range of higher doses of the *galU *mutant (1 × 10^5^-1 × 10^7 ^CFU; n = 4) or WT FT (5 × 10^4 ^CFU; n = 5). Statistical analysis of survival curves was performed using Gehan-Breslow-Wilcoxon tests and p values of 0.027 (*) and 0.009 (**) are indicated. Results shown are representative of two experiments of similar design.

To determine whether the *galU *gene is important for FT virulence, C57Bl/6J mice (5/group) were inoculated intranasally with 5 × 10^4 ^CFU (50 × LD_50_) of either the *galU *mutant or WT FT and then were monitored for 15 days. Each of the mice challenged with the *galU *mutant experienced transient weight loss but survived and completely cleared the infection, while all of the mice challenged with WT FT lost weight continually until they succumbed to tularemia (Figure 2A and 2B). An additional challenge trial in which C57Bl/6 mice (4/group) were challenged with higher numbers of the *galU *mutant (up to 10^7 ^CFU) revealed that this mutant is highly attenuated, with an LD_50 _that is at least 5 logs higher than that of WT FT (Figure 2C). Moreover, trans-complementation of the *galU *mutation completely restored virulence of the mutant strain (Figure 2A). These findings indicated that FT virulence in mice is dependent on the expression of a functional *galU *gene product.

**Figure 3:**
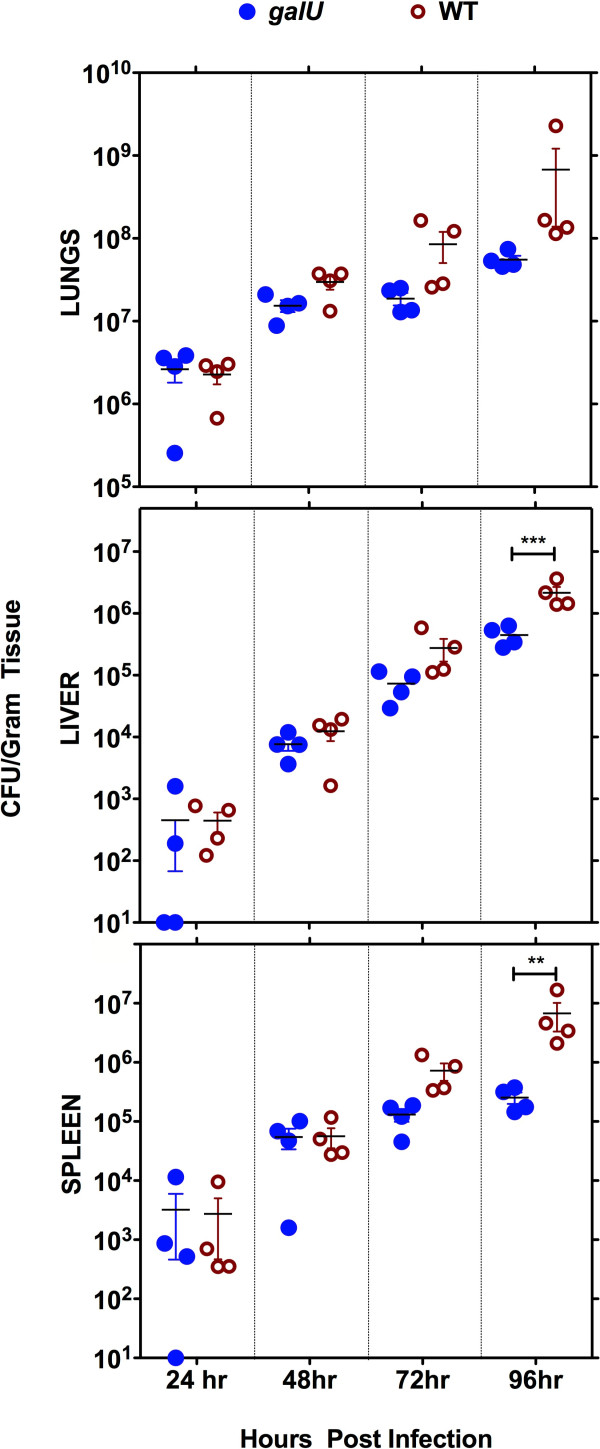
**Mutation of the *galU *gene does not attenuate infectivity of FT *in vivo***. C57BL/6 mice (4/group) were infected intranasally with 5 × 10^4 ^CFU (50 × LD_50 _for FT LVS) of either the WT or *galU *mutant strain of FT LVS. Organs were harvested at 24, 48, 72 and 96 hours p.i. and CFU/g of organ was determined for lungs, liver, and spleen. The lower limit of detection was 20 CFU/g. Statistical analyses were performed via two-way ANOVA with a Bonferroni multiple comparisons post test and all significant differences are indicated as follows: ** P < 0.01 and *** P < 0.0001. The data shown is representative of two independent experiments of similar design.

To determine whether the reduced virulence of the *galU *mutant was the result of defective replication and/or dissemination of the bacterium *in vivo*, we performed a kinetic analysis of bacterial burdens following infection. C57Bl/6J mice (16/group) were challenged with 5 × 10^4 ^CFU of either the *galU *mutant or WT FT and then four mice were sacrificed at each time point (24, 48, 72, and 96 h post-infection) for bacterial burden determinations from the lungs, livers, and spleens (Figure 3). The bacterial burdens observed in the *galU *mutant- and WT-infected mice were similar in each of the tissues for the first 48 h, indicating that mutation of *galU *did not confer any significant defects in replication or dissemination of FT *in vivo*. However, the burdens observed in the *galU *mutant-infected mice were significantly lower (*p *< 0.01) in the spleens and livers (*p *< 0.001) of infected mice at the 96 h time point. Collectively, these results reveal that despite its normal replication/dissemination phenotypes, the *galU *mutant is more readily cleared than WT FT.

### Mutation of *galU *alters the kinetics of innate immune responses

**Figure 4:**
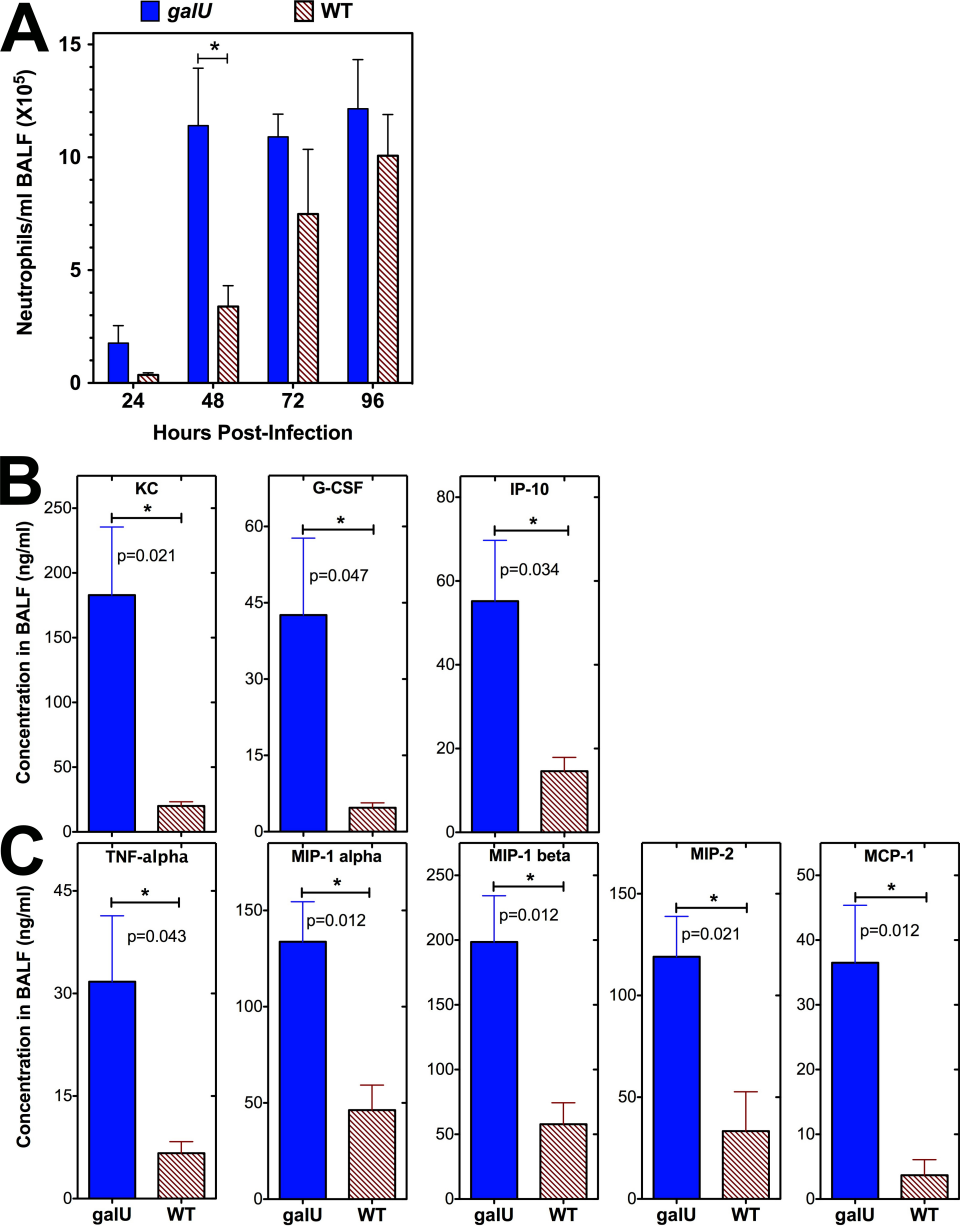
**Neutrophil recruitment and chemokine expression in the lungs following infection with the *galU *mutant**. C57Bl/6J mice (4/group) were infected intranasally with 5 × 10^4 ^CFU (or 50 × LD_50_) of either the WT or *galU *mutant strain of FT and BALF was collected from individual mice at 24, 48, 72 and 96 hours post-infection. Flow cytometric analyses were performed on the cells recovered from BALF to determine the numbers of neutrophils at each timepoint. Statistical analyses were performed via two-way ANOVA with a Bonferroni multiple comparisons post-test and statistically significant differences (P < 0.05) are indicated (*) (**Panel A**). The concentrations of KC, G-CSF, MIG, and IL-10 (**Panel A**) and TNF-α, MIP-1α, MIP-1β, MIP-2, and MCP-1 (**Panel B**) in BALF at the 24 and 48 hour time points, respectively, were determined using a Luminex multiplex kit. Statistical analyses were performed using unpaired t tests. Statistically significant differences (*) and p values are indicated in each panel. The data shown is representative of three independent experiments of similar design.

To determine whether differences in innate immune recognition of infection might be responsible for the dramatic difference in the outcome of disease with the *galU *mutant vs. WT FT, we analyzed the kinetics of immune cell infiltration into the lungs following infection. BALF were collected from each mouse at the time of sacrifice and a series of flow cytometric analyses was performed. The numbers of macrophages, dendritic cells, and NK cells recruited into the lungs of mice infected with the *galU *mutant and WT FT were similar at each time point (data not shown). However, higher numbers of neutrophils were observed in the lungs of mice infected with the *galU *mutant at the 24- and 48-hour time points, with peak numbers of neutrophils measured at 48 hours post-infection (Figure 4A). In contrast, the kinetics of recruitment of neutrophils into the lungs of mice infected with WT FT was much slower (Figure 4A), peaking five days post-infection (data not shown).

Using a Luminex multiplex kit, we also measured the levels of a panel of cytokines/chemokines in the BALF collected from each mouse and found that the levels of several neutrophil chemoattractants CXCL1**/**KC [35], granulocyte colony stimulating factor or G-CSF [36], CXCL10/IP-10 [37], TNF-α [38], MIP-1α/CCL3 and MIP-1β/CCL4 [39], CXCL2/MIP-2 [40], and CCL2/MCP-1 [41] were all present at significantly higher levels in the lungs of *galU *mutant-infected mice (*p *< 0.05) at the 24 or 48 h time points (Figure 4B and 4C), correlating well with the peak of neutrophil recruitment at 48 h post-infection. The levels of these same chemokines/cytokines peaked in the lungs of WT FT-infected mice 72-96 hours post-infection (data not shown), corresponding well with the peak of neutrophil recruitment into the lungs on day five post-challenge.

**Figure 5:**
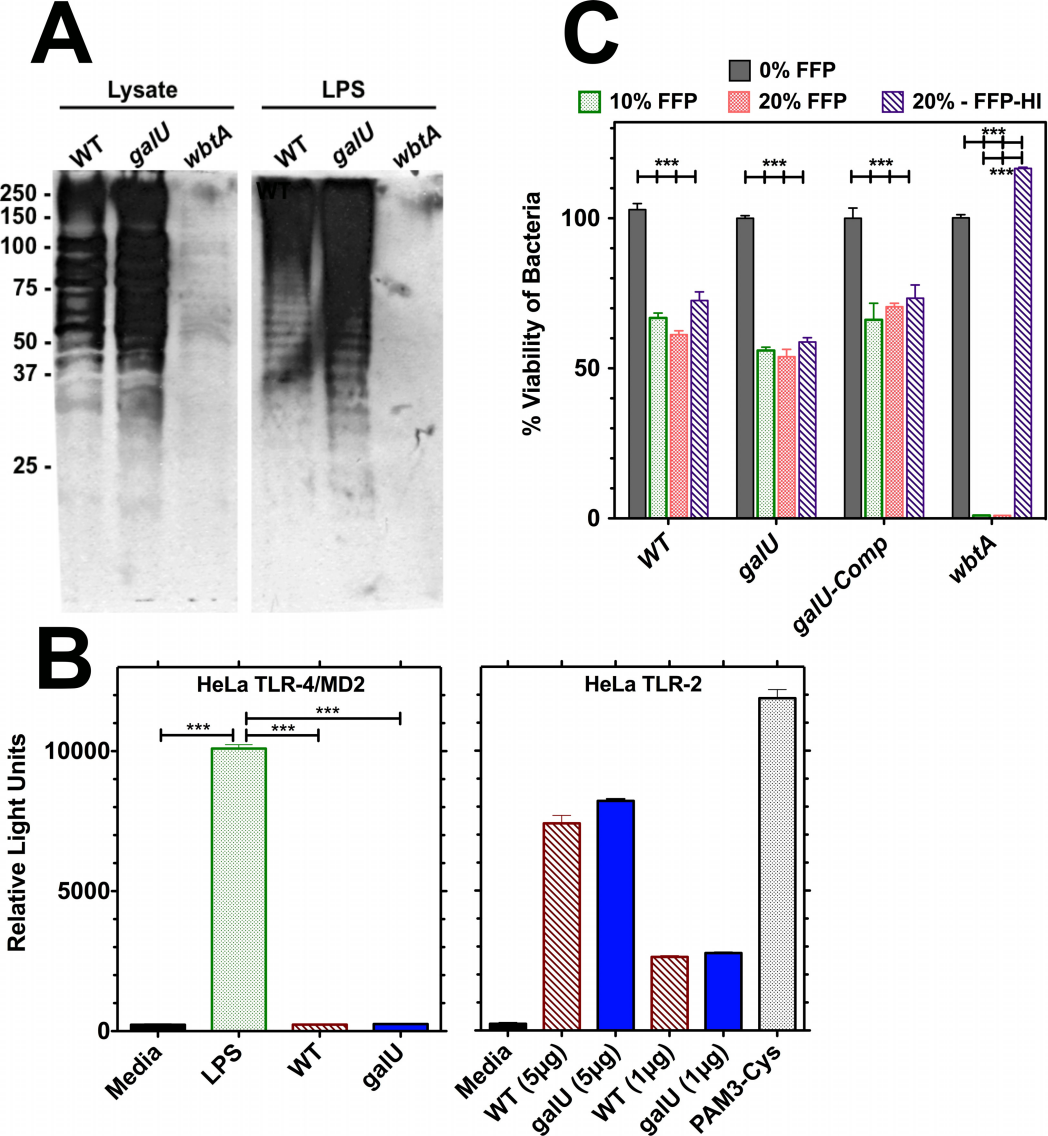
**Mutation of *galU *does not cause gross changes in O-antigen synthesis, serum sensitivity, or TLR signaling**. **Panel A: **Bacterial cell lysates (10 μg/lane) and LPS preparations of WT, *galU *mutant, and *wbtA*-mutant (O-antigen deficient) FT strains were subjected to SDS-PAGE and Western blotting using an FT LPS-specific monoclonal antibody preparation. **Panel B**: HeLa-TLR4/MD-2 or HeLa-TLR2 were transiently transfected with a ELAM-luciferase reporter construct, CMV-CD14 and CMV-β-Gal (for normalization) and stimulated for 6 hours with 2μg or 10μg of the indicated FT lysates. NF-κB activation was measured via a luciferase assay. Statistical analyses were performed via one-way ANOVA and significant differences (P < 0.0001) are indicated (***). **Panel C**: Bacteria were incubated for 2 h at 37°C in either PBS alone or PBS containing either 10% or 20% fresh frozen human plasma (FFP) or 20% heat-inactivated (HI-FFP). Viable bacteria were then enumerated by dilution plating. 100% viability was defined as the number of bacteria recovered from PBS containing no serum (0% FFP), and results were plotted as the mean (± SEM) of triplicate samples. Statistical analyses were performed via one-way ANOVA and statistically significant differences (P < 0.0001) are indicated (***).

It was recently reported that mutations that result in alterations in LPS structure, making the bacterium more likely to be recognized through TLR4 signaling, could result in robust chemokine expression and early neutrophil recruitment [17,20]. To determine if the altered kinetics of innate immune responses observed for the *galU *mutant strain resulted from gross alterations to its LPS structure, we extracted LPS from WT, *galU *mutant, and *wbtA *mutant (O-antigen deficient) strains of FT and performed Western blot analysis using a FT LPS-specific mAb. No obvious alteration in LPS laddering was observed, suggesting that mutation of *galU *did not result in gross changes in synthesis of the O-antigen component of LPS (Figure 5A). We also analyzed the ability of LPS derived from the *galU *mutant to initiate TLR4-mediated signaling. Using HeLa cells that stably express either TLR2 or TLR4/MD2 that had been transfected with a vector bearing a NFκB-responsive luciferase reporter construct, we determined that neither *galU *mutant or WT FT lysates were able to stimulate TLR4 while both stimulated TLR2 to the same extent (Figure 5B), suggesting that the lipid A portion of the mutant LPS was not altered.

To further investigate whether the *galU *gene resulted in gross change(s) to the outer envelope of FT, experiments were performed to measure the relative sensitivity of *galU *mutant and WT FT to serum components. The *galU *mutant, WT, and *galU*-complemented strains of FT all displayed a similar pattern of serum sensitivity. In contrast, an O-antigen-deficient (Δ*wbtA *mutant) strain of FT was highly sensitive to serum. Interestingly, the *galU*, WT, and *galU*-complemented strains were equally sensitive to heat-inactivated serum, while the *wbtA *mutant strain displayed no sensitivity to serum that had been heat inactivated (Figure 5C).

### IL-1 expression/activation induced by the *galU *mutant vs. WT FT

**Figure 6:**
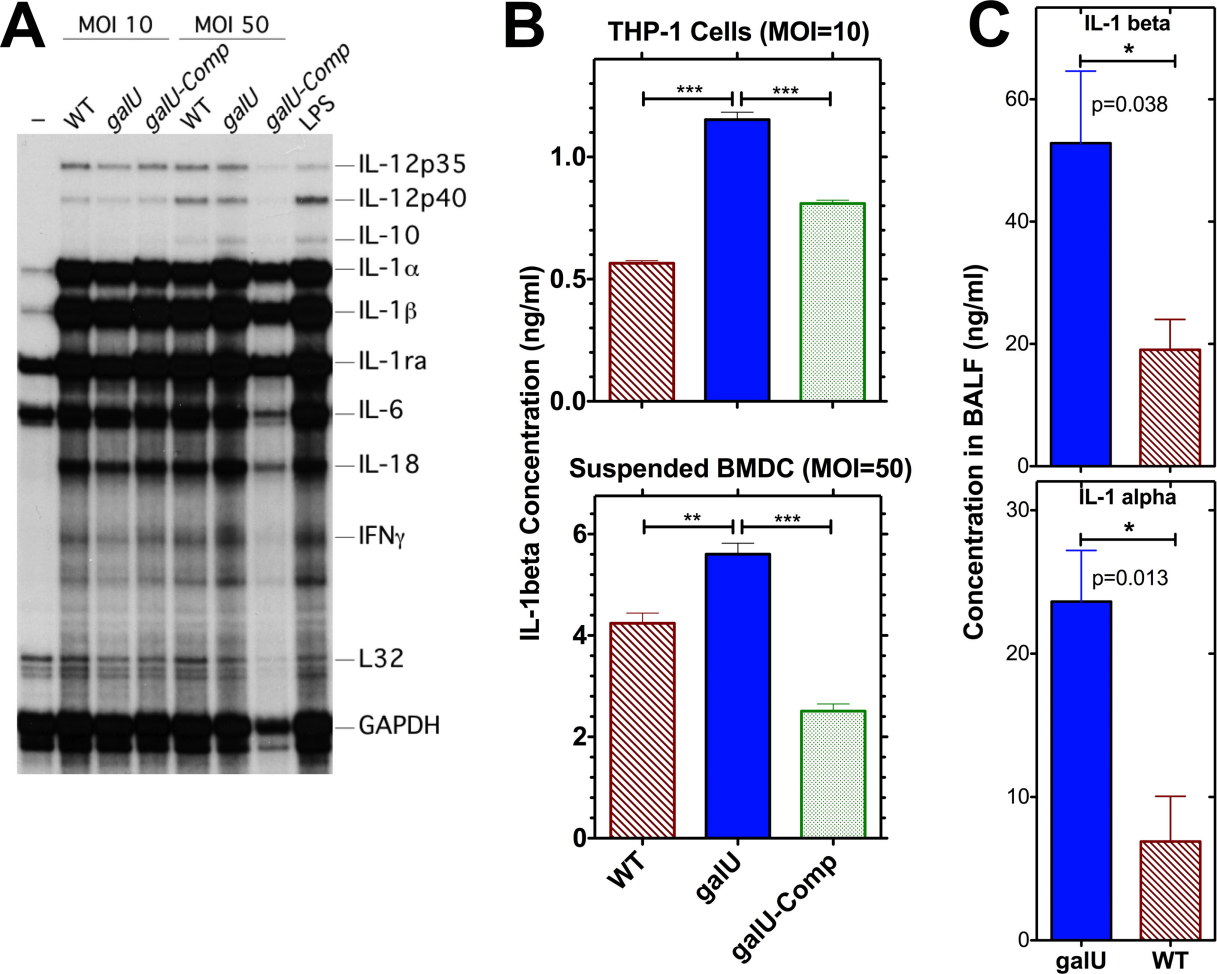
***galU *mutant and WT FT differentially induce cleavage of pro-IL-1β to the active IL-1β form**. **Panel A**: BMDC were infected with WT, *galU *mutant, and *galU*-complemented strains of FT at the indicated MOI, and total RNA was extracted 8 h later and subjected to RNase protection analysis. **Panel B**: IL-1β concentrations in culture supernatants from THP-1 and BMDC following infection (24 h) *in vitro *with either WT, *galU *mutant, or *galU*-complemented strains of FT were determined via ELISA, and statistical analyses were performed via one-way ANOVA with a Bonferroni multiple comparisons post-test (p values are indicated as follows: ** P < 0.01, and *** P < 0.0001). **Panel C**: IL-1β and IL-1α concentrations in BALF collected from mice 48 hrs following intranasal infection with either WT or *galU *mutant strains of FT were determined via multiplex cytokine analysis. Statistical analyses were performed via unpaired t tests and two-tailed p values are indicated.

Activation of the AIM2 inflammasome and production of IL-1β and IL-18 are known to be a critical component of the innate immune response to FT infection [42]. We compared the kinetics of IL-1β production following infection (*in vitro *and *in vivo*) with either the *galU *mutant or WT strain of FT. RNase protection analysis revealed that IL-1β mRNA levels (as well as those of several other cytokines) were similar in bone marrow-derived dendritic cells (BMDC) that had been infected for 8 h with either the *galU *mutant, WT, or *galU*-complemented strains of FT (Figure 6A), confirming the comparable abilities of the *galU *mutant and WT strains to stimulate TLR-mediated events such as cytokine expression. However, 24 h after infection of a macrophage-like cell line (THP-1) or BMDCs with the *galU *mutant, the amount of IL-1β released into culture supernatants was significantly higher (*p *< 0.0001 and *p *< 0.01, respectively) than was observed following infection with WT FT (Figure 6B). The *galU *mutant also induced accelerated kinetics of IL-1β protein production *in vivo *(Figure 6C). Moreover, the kinetics of IL-1α protein production is more rapid following infection with the *galU *mutant strain of FT (Figure 6C).

### Cytotoxicity of the *galU *mutant

**Figure 7:**
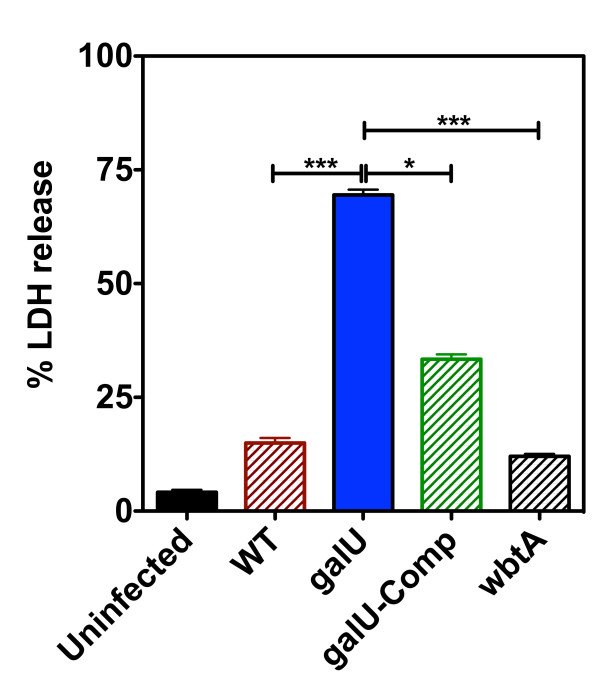
**Mutation of the *galU *gene increases cytotoxicity of FT**. Murine macrophage-like cells (J774) were infected with the WT, *galU *mutant, the *galU*-complemented, or *wbtA *mutant (O-antigen-deficient) FT LVS strains at an MOI of 100. Host cell death was determined by measuring LDH released from infected cells 24-hours post-infection. All data points represent the mean (± SEM) of triplicate samples and the data shown is representative of three experiments of similar design. Statistical analyses were performed via one-way ANOVA with a Bonferroni multiple comparisons post-test (*** indicates a p-value of <0.0001).

In light of the findings that mutation of the *galU *gene resulted in altered kinetics of innate signaling and earlier production of IL-1β than was observed with WT FT, we speculated that the *galU *mutant might induce death of the host cell more rapidly than WT FT. To investigate this possibility, we evaluated the relative abilities of the *galU *mutant and WT FT strains to kill their host cells *in vitro*. A macrophage-like cell line (J774) was infected with either the *galU *mutant or WT FT strains at an MOI of 100 and incubated for 24 hours. LDH activity in the culture medium was then determined as a measure of host cell death. A significantly higher amount of LDH activity was measured in the supernatants of J774 cells that had been infected with the *galU *mutant compared to those infected with WT FT (*p *< 0.0001), indicating that the *galU *mutant was hyper-cytotoxic. Complementation of the *galU *mutation *in trans *partially restored the cytotoxicity phenotype. For comparative purposes, a *wbtA *mutant strain of FT was also included and was shown to have cytotoxicity characteristics similar to those of WT FT (Figure 7).

### Immunization with the *galU *mutant confers immunity to WT FT challenge

**Figure 8:**
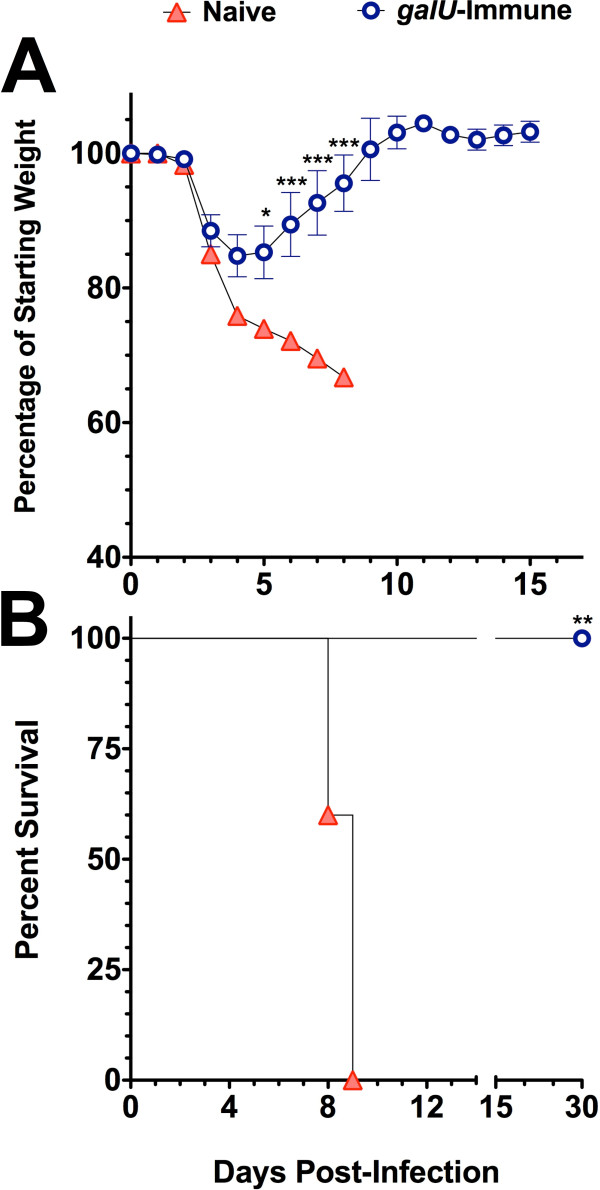
**Infection with the *galU *mutant of FT LVS elicits protective immunity WT FT LVS**. C57Bl/6J mice (n = 5) that had survived intranasal challenge with the *galU *mutant FT strain and naïve control mice (n = 5) were challenged intranasally with 5 × 10^4 ^CFU (50 × LD_50_) of WT FT LVS eight weeks following the initial infection. The body weight (**Panel A**) and survival (**Panel B**) of mice were monitored for survival for 30 days. Statistical analyses of changes in body weight were performed via two-way ANOVA using a Bonferroni multiple comparisons post-test and p-values are indicated as follows: * P < 0.05 and *** P < 0.001. Statistical analysis of the survival data was performed using a Gehan-Breslow-Wilcoxon test (** indicates a p-value of 0.0043).

Because infection with the *galU *mutant elicited a robust innate immune response and infected mice were able to clear the infection, we assessed the efficacy of the *galU *mutant strain as a live attenuated vaccine strain. Two months following the initial inoculation, mice that survived infection with the *galU *mutant, as well as a naïve group of mice, were challenged with a large dose of WT (50 × LD_50_) via the intranasal route and were monitored for survival. The *galU *mutant-immunized mice experienced transient weight loss following challenge, but displayed no other visible symptoms of tularemic disease and survived the infection. In contrast, each of the naive mice displayed the typical visible signs of tularemia (lack of grooming, hunched posture, reduced motor activity, etc.) and succumbed to WT FT infection by day 8 post-challenge (Figure 8).

## Discussion

A major focus of FT research continues to be the identification of virulence-mechanisms used by this extremely virulent pathogen. A number of virulence determinants have been identified, but there remains much to discover regarding the virulence mechanisms used by FT to survive and cause disease within its mammalian hosts. In this report we show that mutation of *galU *results in a dramatic attenuation of FTLVS virulence that appears to be unrelated to any *in vivo *infectivity or growth defects. Although it is known that mutation of the *galU *gene leaves some other bacterial pathogens attenuated for virulence [27,32,43,44], this is the first report examining the role of *galU *in the pathogenesis of FT.

Neutrophils are a critical component of the innate immune responses to bacterial infection, and the recruitment of these cells into the lungs following pneumonic infection typically peaks by 48-hours post-infection [45-47]. However, it has been reported elsewhere [22,25] and confirmed here that neutrophil recruitment following wild type FT infection in the lungs is not detected until approximately 72 h post-infection. Because it is known that neutrophils are required for control of FT infection [48], it is reasonable to speculate that the ability of FT to delay the kinetics of neutrophil recruitment into the lungs following pulmonary infection may be an important virulence determinant. Interestingly, a comparative analysis following pulmonary infection of mice with the *galU *mutant and WT strains of FT revealed that the kinetics of neutrophil recruitment (and production of chemokines/cytokines involved in neutrophil recruitment) occurs much more rapidly following infection with the *galU *mutant (peaks at 48 h post-infection). Kinetic analyses of bacterial burdens in the lungs, spleens, and livers of mice following infection with the *galU *mutant and WT strains of FT revealed that the two strains disseminated and replicated at comparable rates, but the bacterial burdens in *galU*-infected animals became significantly lower than in WT-infected animals by 72 h post-infection. The significant difference in bacterial burdens observed in *galU *mutant- vs. WT FT-infected mice 3-4 days post-infection may have been (at least in part) due to differences in the kinetics of neutrophil recruitment.

It remains unclear whether FT actively suppresses innate immune responses during the early stages of infection, or if the delayed response is due to poor recognition of FT through host pattern recognition receptors. It has been well documented that FT produces an atypical LPS that is not recognized via TLR4 [49-51] and that FT is recognized via the TLR2 signaling pathway [52-55]. Because the *galU *gene has been shown to be important for LPS production [27,31,32,43,56] in a number of other bacterial systems, we performed a series of studies to determine whether differences in the LPS expressed by the FT *galU *mutant might contribute to its reduced virulence. A western blot of both bacterial extracts and LPS preparations revealed no obvious differences in the O-antigen laddering between the *galU *mutant and WT strains of FT, suggesting that mutation of *galU *did not have any gross effects on O-antigen synthesis. Because it has been reported elsewhere [57] and confirmed here (*wbtA *mutant) that the absence of O-antigen is a major determinant of susceptibility to complement-mediated killing, our findings that the *galU *mutant displayed a WT serum sensitivity phenotype also suggested that O-antigen synthesis was not significantly altered by mutation of the *galU *gene. This finding contrasted with reports that *galU *mutant strains of *P. aeruginosa *and *V. cholerae *displayed increased serum sensitivity [31,44]. We also observed no differences between the *galU *mutant and WT strains of FT with respect to signaling via the TLR2 and TLR4 recognition pathways. It remains possible that mutation of *galU *results in minor O-antigen compositional changes, alterations in the core oligosaccharides, or differences in the carbohydrate modification of surface proteins of FT. Moreover, in light of the published finding that mutations causing alterations in the lipid A of FT *novicida *[17,20] are highly attenuating for virulence *in vivo *(possibly due to altered kinetics of cytokine/chemokine production and neutrophil mobilization), we posit that mutation of the *galU *gene may have an impact on the lipid A moieties of FT. A complete analysis of the carbohydrate components of the FT *galU *mutant is needed to identify such differences.

Recent studies have revealed that the innate immune response to FT infection is complex and involves multiple signaling pathways. Others and we have previously shown that FT elicits a powerful inflammatory response that is primarily mediated by TLR2 and caspase-1 activation [52-55]. More recently, it has been demonstrated that the AIM-2 inflammasome mediates caspase-1 activation and secretion of mature IL-1β and IL-18 during FT infection [42,58,59]. We predicted that the ability of the *galU *mutant strain of FT to accelerate the kinetics of inflammatory cytokine production/neutrophil mobilization *in vivo *might be due to more rapid activation of the inflammasome. To address this hypothesis, we measured IL-1β protein production by either THP-1 cells or BMDCs infected for 24 h *in vitro *and found that the *galU *mutant induced higher concentrations of IL-1β than did WT FT. However, RNase protection assays revealed that the differences in IL-1β production by *galU *mutant- vs. WT FT-infected cells were not the result of differential transcription of the IL-1β gene and, therefore, were likely due to more robust activation of the inflammasome. Our findings that production of IL-1β (as well as IL-1α) was induced significantly earlier in the lungs of *galU *mutant vs. WT FT-infected mice were also consistent with the hypothesis. Moreover, we showed that macrophage-like J774 cells infected *in vitro *with the *galU *mutant are killed more rapidly than those infected with WT FT and that WT cytotoxicity could be partially restored by complementation *in trans *with the *galU *gene. These findings were consistent with the possibility that the *galU *mutant more rapidly activates the inflammasome that, in turn, initiates host cell death via pyroptosis and limits the ability of the bacteria to replicate [60]. Based on findings with other mutant strains that display a hypercytolytic phenotype [61,62], it could be speculated that such a change in the *in vivo *life cycle of FT could result in significant attenuation of virulence like that observed for the *galU *mutant. Overall, the findings shown here with FTLVSΔ*galU *are consistent with recently published studies showing that mutation of either *mviN *(FTL_1305 [63]) or *ripA *(FTL_1914 [64]) results in attenuated FT strains that activate the inflammasome more efficiently. Additional studies designed to delineate the signaling pathway(s) that enable early inflammasome activation by the *galU *mutant strain of FT are warranted.

Because the *galU *mutant was so severely attenuated for virulence, in spite of its normal ability to replicate and disseminate *in vivo*, and because there still is no well-defined and efficacious vaccine for FT, we performed a vaccine trial with the *galU *mutant strain. Mice that had been infected with the *galU *mutant and had survived the infection were challenged intranasally two months later with a large dose (50 × LD_50_) of WT FT LVS and all were found to be immune to FT. These findings, coupled with the fact that the *galU *gene is 100% conserved between the LVS and Schu S4 strains, suggest that a *galU *mutant strain in the Schu S4 background could have strong prophylactic potential as a live attenuated vaccine strain. Studies to characterize *galU *in FT SchuS4 are currently underway in our laboratory.

## Conclusions

Disruption of the *galU *gene of FTLVS has little if any effect on its infectivity, replication, or dissemination *in vitro*, but it resulted in highly significant virulence attenuation. The reduced virulence of the *galU *mutant appears to be related to its increased ability to induce innate immune responses following infection. Inflammatory responses and chemokine/cytokine production elicited by WT FT proceeds with much slower kinetics than typically observed for other bacterial pathogens. In contrast, the kinetics of chemokine/cytokine expression and neutrophil recruitment is more rapid following infection with the *galU *mutant strain, likely resulting in more rapid uptake and killing of bacteria by neutrophils. These studies also revealed that disruption of the *galU *gene results in a hypercytotoxic phenotype that could be due (at least in part) to activation of the AIM-2 inflammasome. The accelerated death of cells infected with the *galU *mutant strain presumably interferes with the normal replicative cycle of the bacterium, resulting in the significant difference in bacterial burdens in the liver and spleen of mice infected with the *galU *mutant vs. WT strains of FTLVS observed 4 days post-infection and contributing to the reduction in FTLVSΔ*galU *virulence. These findings underscore the need for studies designed to understand the mechanisms used by WT FT to alter the kinetics of innate immune responses following infection. A thorough comparative analysis of the outer envelope of the WT and *galU *mutant strains of FTLVS coupled with a more detailed analysis of the innate signaling that results following infection with these two strains of FT could lead to a better understanding of the ability of FT to avoid detection by the innate immune system during the early stages of infection. The findings presented here also suggest that a *galU *mutant strain of FT has high potential as a platform for development of a live attenuated tularemia vaccine strain.

## Methods

### Bacteria and Culture Conditions

FTLVS was a kind gift of Dr. Karen Elkins (FDA, Bethesda, MD). The FTLVS *galU *mutant strain was identified by screening a LVS transposon mutant library for mutants exhibiting elevated susceptibility to polymyxin B. Transposon insertion in to the *galU *gene was verified by DNA sequencing and the polymyxin B hypersensitive phenotype was verified by complementation. The results of this screen will be described in a future publication. FT strains were grown at 37°C in Mueller-Hinton (DIFCO/Becton Dickinson, Sparks, MD) broth modified with 2.5% ferric pyrophosphate, 0.1% glucose, and 10% cysteine (MMH). The *galU *mutant was grown under kanamycin selection (10 μg/mL). Complementation studies were performed as follows. The *galU *gene was amplified by PCR from the LVS genome using primers: forward primer: 5'-CTCGTGGATCCGCTAAAATGAAAATAAGAAAAGC-3' and reverse primer: 5'-ATCGCTAATCGATAAGCTATCTATTTTGAAGG-3'. The resulting amplicon was digested with BamHI and ClaI restriction endonucleases before being ligated to similarly digested pXB167 [65], which placed the *galU *gene downstream and in the same orientation as the constitutively expressed *orf5 *promoter. The resulting plasmid, pXB167-*galU*, was then introduced into the indicated strains by electroporation as previously described [15,65]. The *galU*-complemented strain was grown in presence of kanamycin (10 μg/mL) and carbenicillin (350 μg/mL). Bacterial stocks were made from overnight cultures of bacteria grown to OD_600 _of 0.7-0.9, and aliquots were frozen at -80°C.

### Antimicrobial Susceptibility Determination

Antimicrobial susceptibility was determined by the gradient agar plate method [15,66]. The gradient agar plates were prepared in 90 mm × 90 mm Petri plates as follows. Thirty-five milliliters of BHI-chocolate agar (without the test compound) was poured into the square Petri dish and allowed to harden as a wedge by elevating one side of the plate. After the agar solidified, 35 mL of BHI-chocolate agar containing the test compound were added to the leveled plate and allowed to solidify. The antibiotic gradient plates were allowed to develop for 2 h and inoculated within 3 h after preparation. Growth was measured after two days of incubation at 37°C. All tests were performed in triplicate. Minimal inhibitory concentrations (MIC) were determined as follows: MIC = distance of growth (mm) × concentration of drug (ex. μm/mL)/90 (mm).

### Mice

C57BL/6 mice were purchased from Charles River Laboratories. Mice were age-matched and used between 8 and 16 weeks of age. Mice were housed in microisolator cages with food and water available *ad libitum*. All experimental protocols were reviewed and approved by the University of Tennessee Health Science Center IACUC.

### Intranasal Challenge of Mice with FT

Mice were lightly anesthetized using isoflurane administered with a Vapor Stick nebulizer. Frozen stocks of FT were thawed anew for each experiment, diluted in phosphate-buffered saline (PBS), and administered intranasally (20 μl/naris). The CFUs of FT in the inocula were verified by dilution plating. Following challenge, all mice were monitored daily for signs of illness (decreased mobility, ruffled fur, hunched gait) and weight loss. Upon sacrifice, bronchoalveolar lavage was performed and spleens, livers and lungs were collected for bacterial burden assessment.

### Bacterial Burden Determination

Spleens, livers and lungs of challenged mice were removed aseptically and homogenized (using a tissue homogenizer) in one milliliter of sterile PBS. To disrupt cells (releasing FT), 0.25 mL disruption buffer (2.5% saponin, 15% BSA, in PBS) was added with light vortexing. Appropriate dilutions of each sample were then plated in duplicate using an Eddy Jet spiral plater (Neutec Group Inc., Farmingdale, NY) on MMH agar plates (supplemented with 5% calf serum) and incubated at 37°C for 48-72 hours. Colonies were counted using a Flash & Go automated colony counter (Neutec Group Inc.).

### Cell Culture, Macrophage Infection, and Cytotoxicity Assays

J774 and RAW264.7 cells (ATCC) were propagated in Dulbecco's Modified Eagle's Medium (DMEM) containing 10% fetal bovine serum. For replication assays, cells were seeded in 24 well tissue culture plates at a density of 2 × 10^5 ^cells per well. Twenty-four-hours later, FT was added (MOI of 10) and incubated for 2 h. Gentamicin was then added (50μg/mL) and incubated for 1 hour to kill extracellular bacteria. Cells were then washed two times with DMEM and incubated in fresh culture medium at 37°C. At each experimental time point, cells were washed with PBS to remove any bacteria released during the incubation period, lysed in PBS containing 0.1% deoxycholate, and the number of viable bacteria released from the cells was determined via dilution plating.

For cytotoxicity (LDH) assays, J774 cells were seeded into a 96 well plates and allowed to adhere overnight. FT was added to wells (MOI of 100) and the plates were centrifuged (800 × g, 5 min) to facilitate contact between the cells and bacteria. After 2 hours of co-culture with bacteria, the culture supernatant was aspirated and replaced with fresh media containing gentamicin (50μg/mL) and the plates were incubated at 37°C, 5%CO_2 _for 24 hrs. Culture supernatants were then analyzed for LDH release using the CytoTox Non-Radioactive Cytotoxicity Assay (Promega) according to the manufacturer's protocol. The total LDH release (100% LDH in cells) was determined by lysis of uninfected cells. The background LDH value was defined as the level of LDH in the supernatants collected from intact uninfected cells. The percentage of LDH release was calculated as follows: (Sample LDH value - background LDH value)/(Total LDH release value - Background LDH release value) × 100.

Mouse bone marrow-derived dendritic cells (BMDC) were generated by incubating bone marrow in RPMI 1640-10%FCS supplemented with rmGM-CSF (20 ng/mL) (R&D Systems, Minneapolis, MN) for 8 days. This procedure routinely results in 60-80% CD11c^+ ^cells.

### Bronchoalveolar Lavage (BAL) and Flow Cytometric Analysis

BAL was performed as described previously [45]. Briefly, BAL was performed by intratracheal injection of 1 mL of PBS into the lungs with immediate vacuum aspiration. The amount of fluid (BALF) recovered was routinely around 800 μl. Cells were recovered from BALF by centrifugation and their viability was determined by trypan blue exclusion. Protease inhibitor cocktail (Pierce, Rockford, IL) was added to the BALF immediately after recovery and the BALF was frozen at -80°C till further use.

Flow cytometry was performed on isolated BAL cells using fluorochrome conjugated antibodies specific for CD45, CD11b, F4/80, GR1, and NK1.1 (eBioscience CA, USA). A minimum of 50,000 events/sample was collected on a BD Biosciences LSRII cytometer (BD Biosciences, San Jose, CA). Expression of cell surface markers was analyzed using DIVA software. The percentage of neutrophils was determined using gates set on live cells and CD45 expression, and neutrophils were identified as CD11b^high ^/Gr1^high^. Dendritic cells and NK cells were identified as CD11b^high^/GR1^lo^/F480^lo ^and CD45^high^/NK1.1^high^, respectively.

### Chemokine/Cytokine Measurements from BALF

The concentrations of each of the chemokines/cytokines from BALF were determined via multiplex analysis using a Luminex Milliplex Analyzer (Millipore Corp., Billerica, MA). A 32-plex Milliplex Cytokine/Chemokine Immunoassay (Millipore) was used according to manufacturer's instructions to simultaneously measure the following: eotaxin, G-CSF, GM-CSF, IFN-γ, IL-1α, IL-1β, IL-2, IL-3, IL-4, IL-5, IL-6, IL-7, IL-9, IL-10, IL-12 (p40), IL-12 (p70), IL-13, IL-15, IL-17, IP-10, KC, LIF, LIX, MCP-1, M-CSF, MIG, MIP-1β, MIP-1α, MIP-2, RANTES, TNFα, and VEGF. All determinations were performed with duplicate samples, and data analysis was performed using Luminex xPonent and Milliplex Analyst software packages (Millipore).

### Galactose Sensitivity

FT strains were grown overnight in MHB containing 0.1% glucose and then pelleted, washed and resuspended in PBS. Each strain was then diluted to 5 × 10^7 ^CFU/mL and inoculated in fresh MHB containing either 0.1% glucose or 2% D-galactose as the sole sugar source and incubated at 37°C for 24 hours. Optical density at 600 nm was monitored hourly as a measure of growth.

### LPS Isolation

Bacterial cultures in mid-logarithmic growth phase were pelleted by centrifugation at 4000 rpm for 20 min and then resuspended in PBS. LPS was isolated from the bacteria using LPS extraction kit (Intron Biotechnologies, Boca Raton, FL) as per the manufacturer's directions.

### SDS-PAGE and Western Blotting

Bacterial cell lysates (5 μg/lane) and LPS extracts were electrophoresed on 4-20% gradient polyacrylamide gel and transferred to nitrocellulose membrane. The membrane was then blocked with 5% BSA (in PBS+0.1% Tween-20) and probed with an FT LVS O-antigen-specific mAb (unpublished, see below). Bound antibodies were detected by probing with HRP-conjugated goat anti-mouse secondary antibody (Jackson Research Labs) and visualized by addition of Western Lightning Plus-ECL Enhanced Chemiluminescence substrate (Perkin Elmer, Shelton, CT).

The O-antigen-specific mAb used for the Western analysis was generated as follows: Six-week old female C57/BL6 mice were immunized (i.p.) three times at two-week intervals with 5 × 10^7 ^heat-killed FTLVS. Three weeks later each mouse was challenged/boosted via intraperitoneal inoculation with 10^6 ^live FTLVS. Six weeks later, the FT immune mice with high titer anti-FT IgG were boosted via intraperitoneal injection of 5 × 10^7 ^heat-killed FTLVS. Spleens were removed three days later, and splenocytes were fused with P3 × 63-Ag8.653 plasmacytoma cells as previously described [67]. Thirteen days after fusion, hybridoma cell supernatants were screened via direct ELISA for IgG reactive with sonicated FT-antigen and whole FT bacteria. The O-antigen-specific hybridoma was cloned via limiting dilution and mAbs were purified from culture supernatants via affinity chromatography using protein G-sepharose columns (Pierce/ThermoFisher Scientific, Rockford, IL).

### Sensitivity to Human Serum

Overnight cultures of the indicated FT strains were pelleted via centrifugation at 4000 rpm for 20 min and washed once with PBS. The bacteria (1 × 10^7 ^CFU) were suspended in 100 μl PBS and incubated with an equal amount of human fresh frozen plasma (citrate was used as the anticoagulant) at varying concentrations for 2 h at 37°C. Surviving bacteria were enumerated by dilution plating on MMH plates.

### TLR4/TLR2 Signaling Luciferase Assay

HeLa-TLR4/MD2 or HeLa-TLR2 [68] were transiently transfected in 24-well plates using Effectene reagent (Qiagen) with 0.4μg of ELAM-luciferase, 0.2μg of pcDNA-CD14 and 0.1μg of CMV-β-Gal expression plasmids (recipe for 24 wells). Forty-eight hours after transfection, the cells were stimulated for 6 hours with FT lysates. LPS (10 ng/mL) from ***E. coli ***strain LCD25 (List Biological, Campbell, CA) and PAM3-Cys (1μg/mL; Invivogen, San Diego, CA) were used as controls for TLR4 and TLR2 signaling, respectively. Luciferase assays were performed using Promega (Madison, WI) reagents according to the manufacturer recommendations. Efficiency of transfection was normalized by measuring β-Gal in cell lysates.

### RNase Protection Assays

BMDC seeded into 24-well tissue culture plates (2 × 10^6^/well) were infected with FT and then total RNA was isolated 8 hr later using TRizol reagent (Life Technologies, Grand Island, NY). RNase protection assays were performed with 4μg of total RNA using a BD-Pharmingen (San Diego, CA) Riboquant kit and the mCK-2 multi-probe template set.

### Quantitation of IL-1β Production *In Vitro*

BMDC or THP-1 cells were seeded into 24-well tissue culture plates (2 × 10^6^/well) and infected with FT. Gentamicin was added to the medium 3 hours later. IL-1β was measured in conditioned supernatants 24 hr post-infection using an ELISA kit (eBiosciences, San Diego, CA).

### Statistical Methodology

Statistical analyses of each figure were performed using GraphPad Prism software (GraphPad Software, La Jolla, CA). The specific statistical method used for each dataset is described in the figure legends.

## Competing interests

The authors declare that they have no competing interests.

## List of Abbreviations

FT: *Francisella tularensis*; LVS: live vaccine strain; FTLVS: *Francisella tularensis *live vaccine strain; LD_50_: lethal dose 50; CAMPs: cationic antimicrobial peptides; LPS: lipopolysaccharide; WT: wild type; MHB: Muller-Hinton Broth; MMH: modified Mueller-Hinton Broth; PBS: phosphate-buffered saline (PBS),; BAL: Bronchoalveolar lavage; BALF: bronchiolar lavage fluid; BMDC: bone marrow-derived dendritic cells.

## Authors' contributions

HRJ conceived of and performed most of the experimental work for the study and drafted the manuscript. JP participated in the bulk of the experimental work. EAF participated in and assisted in design of the flow cytometric analyses. JEB and XRB created the transposon library and isolated the *galU *mutant strain of FTLVS. FR assisted in design of and performance of RNase protection and IL-1β measurements from infected cells in vitro. FDE performed the antimicrobial sensitivity assays. MAM oversaw the design and coordination of all studies, performed the statistical analyses, and helped to draft the manuscript. All authors have read and approved the final manuscript.
